# The efficacy and safety of *Baoji Tablets* for treating common cold with summer-heat and dampness syndrome: study protocol for a randomized controlled trial

**DOI:** 10.1186/1745-6215-14-440

**Published:** 2013-12-21

**Authors:** Rui-zhi Feng, Jian-qin Lv, Angela K Johnson, Juan D Montoya, Bing Mao

**Affiliations:** 1 Department of Integrated Traditional and Western Medicine, West China Hospital of Sichuan University, 37 Guoxue Lane, Chengdu, 610041, Sichuan Province, People’s Republic of China; 2 Department of Emergency Medicine, Carolinas Medical Center, 1000 Blythe Blvd, Charlotte, NC 28203, USA; 3 Department of Emergency Medicine, University of North Carolina Hospitals, 170 Manning Drive, Chapel Hill, NC 27514, USA

**Keywords:** Common cold, Summer-heat and dampness syndrome, *Baoji Tablets*, Traditional Chinese medicine, Randomized controlled trial

## Abstract

**Background:**

Despite the high incidence and the economic impact of the common cold, there are still no effective therapeutic options available. Although traditional Chinese medicine (TCM) is widely used in China to treat the common cold, there is still a lack of high-quality clinical trials. This article sets forth the protocol for a high-quality trial of a new TCM drug, *Baoji Tablets*, which is designed to treat the common cold with summer-heat and dampness syndrome (CCSDS). The trial is evaluating both the efficacy and safety of *Baoji Tablets.*

**Methods/design:**

This study is designed as a multicenter, phase II, parallel-group, double-blind, double-dummy, randomized and placebo-controlled trial. A total of 288 patients will be recruited from four centers. The new tablets group are administered *Baoji Tablets* 0.9 g and dummy *Baoji Pills* 3.7 g. The old pills group are administered dummy *Baoji Tablets* 0.9 g and *Baoji Pills* 3.7 g. The placebo control group are administered dummy *Baoji Tablets* 0.9 g and dummy *Baoji Pills* 3.7 g. All drugs are taken three times daily for 3 days. The primary outcome is the duration of all symptoms. Secondary outcomes include the duration of primary and secondary symptoms, changes in primary and secondary symptom scores and cumulative symptom score at day 4, as well as an evaluation of treatment efficacy.

**Discussion:**

This is the first multicenter, double-blind, double-dummy, randomized and placebo-controlled trial designated to treat CCSDS in an adult population from China. It will establish the basis for a scientific and objective assessment of the efficacy and safety of *Baoji Tablets* for treating CCSDS, and provide evidence for a phase III clinical trial.

**Trial registration:**

This study is registered with the Chinese Clinical Trial Registry. The registration number is ChiCTR-TRC-13003197.

## Background

The common cold is the conventional term used to describe a mild, self-limited, viral, upper respiratory tract infection and it is the most prevalent disease experienced by humans [1]. Symptoms usually include a sore or scratchy throat, sneezing, coughing, hoarseness, runny nose, nasal obstruction and mild general symptoms like fever, headache, chills and not feeling well in general [2]. However, in clinical practice, symptoms caused by the common cold vary from person to person, and cold to cold. Well over 200 viruses are implicated in the cause of the common cold [3]. Among these pathogenic viruses, adenoviruses and enteroviruses can even cause diarrhea [4-6]. It has been reported that acute upper respiratory infection is the most common discharge diagnosis in emergency departments and the second most common diagnosis in physician offices [7,8]. In the United States, the costs associated with noninfluenza viral respiratory infections are estimated at 40 billion dollars each year, including indirect and direct costs [9]. Despite the high incidence and the economic impact of the common cold, no effective etiological treatment has been proven. Treatment thus mainly comprises symptomatic relief [10]. Although various treatments are used in clinical practice nowadays, few of them are supported by any solid evidence.

Dissatisfaction with conventional treatments offered by Western medicine has resulted in many patients seeking help from complementary and alternative medicine. Traditional Chinese medicine (TCM), a 5000-year-old ancient system of medicine, has been used to treat a wide range of diseases, including the common cold. The TCM treatment for common cold is based on the differentiation of symptoms and signs, such as fever, fatigue, headache, aversion to cold, nausea and vomiting, nasal discharge, stuffy nose, tongue proper, tongue coating and condition of pulse. With a totally different system of concepts and theories for etiology, TCM divides colds into three categories: wind-heat syndrome, wind-cold syndrome and summer-heat and dampness syndrome. Among them, the common cold with summer-heat and dampness syndrome (CCSDS) is most commonly seen in midsummer and it is primarily characterized by aversion to cold, fever, diarrhea, nausea and vomiting. According to the basic principles of TCM, CCSDS is treated by dispersing summer-heat and resolving dampness, as well as improving the spleen’s transportation function.

A new TCM drug, *Baoji Tablets*, is manufactured by Wanglaoji Pharmaceutical Co Ltd, Guangzhou, China. It is a new dosage form of *Baoji Pills*, which is an old and famous traditional Chinese preparation in the form of tiny round balls, 3 to 5 mm in diameter, used for treating CCSDS. This patented drug is made of active ingredients from the Chinese herbs listed in Table 1, which have all been approved by the China Food and Drug Administration (CFDA) [11]. Preclinical pharmacologic experiments showed that it can disperse summer-heat, resolve dampness and improve the spleen’s transportation function. Furthermore, no evidence of an adverse or toxic effect has been found in toxicological studies. The preclinical studies were completed by Professor Rujun Wang and her research team from the new drug development research center of Guangzhou Traditional Chinese Medical University. Their studies have not yet been published.

**Table 1:** **Standard formulation of *Baoji Tablets***

**Pinyin name**	**Latin name**
Gouteng	*Ramulus uncariae cum uncis*
Juhua	*Flos chrysanthemi*
Jili	*Fructus tribuli*
Houpo	*Cortex magnoliae officinalis*
Cangzhu	*Rhizoma atractylodis*
Tianhuafen	*Radix trichosanthis*
Guanghuoxiang	*Herba pogostemonis*
Gegen	*Radix puerariae*
Huajuhong	*Exocarpium citri grandis*
Baizhi	*Radix angelicae dahuricae*
Yiyiren	*Semen coicis*
Daoya	*Fructus oryzae germinatus*
Bohe	*Herba menthae*
Fuling	*Poria*
Guangdongshenqu	*Massa medicata fermentata*

The objective of this well-designed randomized controlled trial is to evaluate the efficacy and safety of *Baoji Tablets* by comparing it with the commonly used traditional preparation *Baoji Pills*, as well as with a placebo, for patients with CCSDS. In this study, the investigators hypothesize that the duration of all symptoms will be shorter in the new tablets group. Additionally, it is expected that the new drug will be safe for oral administration.

## Methods/design

### Design

**Figure 1:**
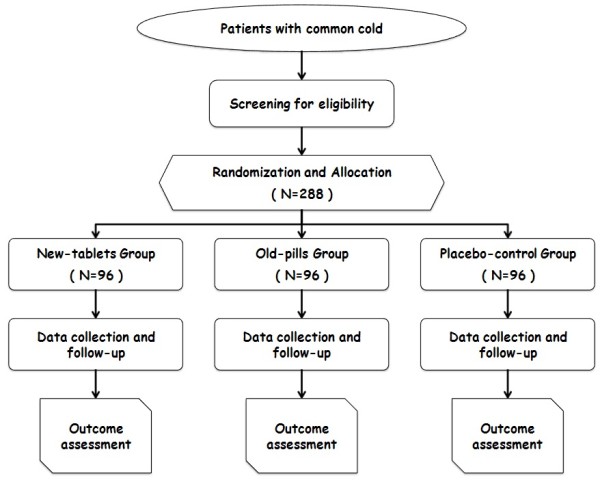
Study flow chart.

This study is designed as a multicenter, phase II, parallel-group, double-blind, double-dummy, randomized and placebo-controlled trial. The trial protocol is conducted in accordance with the Good Clinical Practice Guidelines and the Declaration of Helsinki (2008) [12]. This study has been authorized by the CFDA (Approval No. 2009L00225) and ethics approval has been obtained from local ethics committees, including the West China Hospital of Sichuan University Clinical Trials and Biomedical Ethics Committee, the Ethics Committee of the Affiliated Hospital of Jiangxi College of TCM, the Clinical Research Ethics Committee of Fujian Institute of TCM and the Ethics Committee of Guangxi Traditional Chinese Medical University. In addition, the study is registered with the Chinese Clinical Trial Registry (ChiCTR-TRC-13003197). The study is financially supported by Wanglaoji Pharmaceutical Co Ltd, Guangzhou, China. This funding source had no role in the design of this study and does not have any responsibility for analyses, interpretation of the data or the decision to submit results, other than providing test drugs. Trained research nurses introduce the trial to patients, and give them information sheets and consent forms. All patients have to give their written informed consent prior to enrollment. The study’s flow chart is shown in Figure 1.

### Patient population and setting

A total of 288 Chinese patients, who fulfill the screening criteria, will be enrolled at four large comprehensive hospitals in China: 1) West China Hospital of Sichuan University, 2) the Affiliated Hospital of Jiangxi College of TCM, 3) Fujian Institute of TCM and 4) Ruikang Hospital of Guangxi Traditional Chinese Medical University. Each center will recruit the same number of patients. In other words, 72 patients will be recruited at each hospital. A research assistant in each center will recruit patients through advertisements in local hospitals.

### Western medicine diagnostic criteria for the common cold

First, patients have to answer ‘yes’ to either of the following questions: A) Do you think that you have a cold? B) Do you think you are coming down with a cold? Second, patients have to report at least one of following four symptoms established previously as Jackson’s criteria [13]: 1) nasal discharge, 2) nasal obstruction, 3) sneezing and/or 4) a sore throat.

### Diagnostic criteria for TCM syndrome differentiation

The TCM diagnosis of summer-heat and dampness syndrome is based on the *Guidelines for Clinical Research of New Chinese Medicine*[14]. For a patient to be diagnosed with summer-heat and dampness syndrome, he/she will have at least two of each of the primary and secondary symptoms listed in Table 2, as well as the TCM signs for the tongue and pulse.

**Table 2:** Traditional Chinese medicine diagnostic criteria

**Category**	**Symptoms and signs**
Primary symptoms	Aversion to cold, fever, diarrhea, nausea and vomiting
Secondary symptoms	Fatigue, headache, abdominal discomfort, poor appetite, belching
TCM signs for the tongue	Reddish tongue, greasy tongue coating
TCM signs for the pulse	Floating pulse, soft and slippery pulse

### Inclusion criteria

• Diagnosis of common cold according to Western medicine

• Diagnosis of summer-heat and dampness syndrome according to TCM

• Aged between 18 and 65 years old

• Presents within 48 hours after onset of common cold-like symptoms

• Able to understand and sign written informed consent

### Exclusion criteria

• Patients with acute herpetic pharyngitis, acute viral pharyngitis, acute herpetic laryngitis, acute viral laryngitis, acute conjunctivitis or acute tonsillitis

• Patients with acute bacterial gastroenteritis

• Patients with serious primary diseases of the heart, liver, kidney, blood system or endocrine system

• Patients with acute lower respiratory diseases, tumors or AIDS

• Patients with alanine aminotransferase levels 1.5 times higher than the upper limit of normal reference values, abnormal blood creatinine, positive urine protein qualitative test, blood leukocyte count < 3.0 × 10^9^/L or > 10.0 × 10^9^/L, neutrophil percentage > 80% or stool routine test using a high power objective finds white blood cell count > 1, pus cells or phagocytes

• Patient’s body temperature > 38.5°C

• Patients taking any medication before the study for relief of symptoms

• Pregnant women, hoping to become pregnant women, lactating women

• Patients who have an allergic constitution or are allergic to the trial medicine

• Mentally or legally disabled patients

• Patients who are participating or participated in another drug clinical trial within the prior 3 months

### Other criteria

Participants who meet any of the following criteria are withdrawn from the study:

• Voluntarily quitting

• Misdiagnosis

• Using forbidden drugs or treatments in the course of the trial

• Patient’s actual usage of trial drug is < 80% or > 120% of the required dosage

• Unable to complete the scheduled follow-up

• The patient’s condition worsens during the study and they need hospital treatment

• The patient cannot complete the study due to adverse events or other reasons

Patients who are withdrawn are not replaced.

The whole study will be terminated or suspended early if any of the following occur:

• Serious adverse event

• Poor curative effect is found during the study

• Significant deviation from the protocol

• Flawed protocol

• The pharmaceutical supervisory and administrative department decides to terminate the study for any reason

• The sponsors decide to terminate the trial due to management or funding problems

### Concomitant treatments and forbidden drugs

Use of any other Western therapy or Chinese medicine to relieve symptoms, which may affect the analysis of the final results, is strictly prohibited. Medications used to control other conditions of the participants, such as hypertensive or diabetic medications, are allowed. The dosage, duration and name of any concomitant treatment or medication must be recorded carefully in the case report form (CRF).

### Test drugs

The test drugs are *Baoji Tablets*, dummy *Baoji Tablets*, *Baoji Pills* and dummy *Baoji Pills* and they are manufactured by Wanglaoji Pharmaceutical Co Ltd. Dummy drugs are indistinguishable from the real ones. They are almost the same in shape, size, taste and color. To make blinding more effective, both dummy and real drugs use the same packaging. A uniform test drug package contains both tablets and pills for 3 + 1 days’ dosage. A clearly visible tag on each package states: ‘FOR TRIAL USE ONLY’ and other information, such as the manufacturer’s name, drug name, drug code, usage, dosage and so on. All test drug packages are dispensed and reclaimed by an independent drug administrator in each center, who is also responsible for storing and keeping records of test drugs.

### Randomization and blinding

Stratified blocked randomization is used in this study. Stratification is performed on enrolling centers and block length will be decided by an independent coder, using the software called Package for Encyclopaedia Medical Statistics 3.1 (PEMS 3.1) to generate the randomization sequence. Patients are assigned to the new tablets group, old pills group or placebo control group using a 1:1:1 ratio. The assignment for each patient is concealed in a sealed, lightproof and numbered envelope. Each envelope is assigned to a unique coded test drug package containing the specified medication according to the group assignment. For example, if a patient is assigned to the new tablets group, their unique coded test drug package will contain real *Baoji Tablets* and dummy *Baoji Pills*. Patients are randomly enrolled using the numbered envelopes and assigned to a treatment group. A blinded clinical assistant in each center performs the patient allocation. As the trial is double blinded, both investigators and patients are blinded to group assignments. The sealed envelopes are kept in locked drawers and will only be opened if there is a medical emergency.

### Interventions

#### New tablets group

Patients are administered *Baoji Tablets* 0.9 g and dummy *Baoji Pills* 3.7 g three times daily for 3 days.

#### Old pills group

Patients are administered dummy *Baoji Tablets* 0.9 g and *Baoji Pills* 3.7 g three times daily for 3 days.

#### Placebo control group

Patients are administered dummy *Baoji Tablets* 0.9 g and dummy *Baoji Pills* 3.7 g three times daily for 3 days.

**Figure 2:**
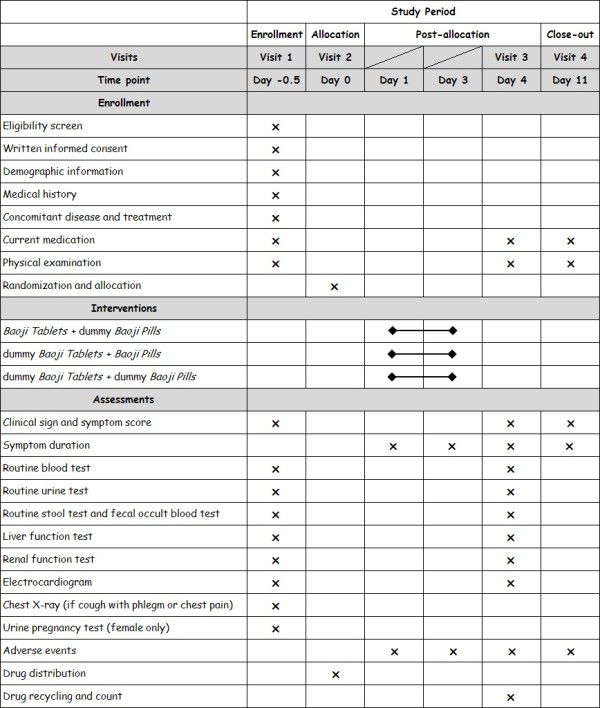
Study schedule for patients.

All groups receive health education for the common cold, such as the recommendation to increase rest and fluid intake. During the study, patients are visited four times by the investigators. The time points for the visits are 0.5 days before baseline, at baseline, 4 days post baseline and 11 days post baseline. The follow-up visits last for one week after the treatment. Details of study visits and contents can be found in Figure 2.

### Outcome measurements

#### Primary outcomes

The primary outcome of this study will be the duration of all symptoms, which is defined as the number of hours from baseline to the time when the patient feels not sick at all. This can be acquired from the patient diary, in which each patient records any changes in symptoms.

#### Secondary outcomes

**Figure 3:**
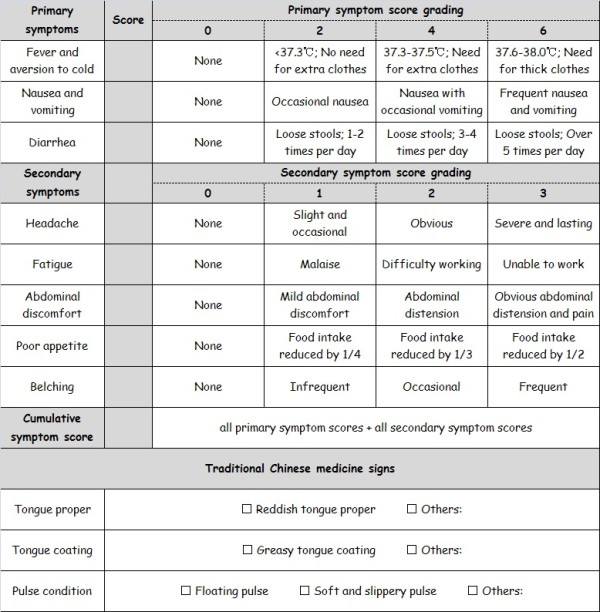
Symptom scores and traditional Chinese medicine signs.

Secondary outcomes include duration of primary and secondary symptoms, changes in primary and secondary symptom scores and cumulative symptom score at day 4, as well as the evaluation of treatment efficacy. In this trial, the symptom score system and treatment efficacy evaluation system follow the *Guidelines for Clinical Research of New Chinese Medicine*[14]. In the symptom score system, the primary and secondary symptoms are given graded scores. The cumulative symptom score is the sum of all symptom scores. In addition, TCM signs will also be assessed (Figure 3). The treatment efficacy evaluation system uses the percentage of symptom score reduction (PSSR). The PSSR for a patient after treatment is calculated according to the following formula:

$$\mathrm{PSSR}=\left(\frac{\mathrm{Symptom}\;\mathrm{score}\;\mathrm{at}\;\mathrm{day}\;0-\mathrm{Symptom}\;\mathrm{score}\;\mathrm{at}\;\mathrm{day}\;4}{\mathrm{Symptom}\;\mathrm{score}\;\mathrm{at}\;\mathrm{day}\;0}\right)\times 100\%$$

The symptom improvement will be classified as full recovery (PSSR ≥ 90%), good recovery (90% > PSSR ≥ 70%), modest recovery (70% > PSSR ≥ 30%) and no recovery (PSSR < 30%).

#### Safety outcomes

Safety outcomes include results of a routine blood test, routine urine test, routine stool test, fecal occult blood test, liver function test, renal function test and electrocardiogram. These biological indicators are monitored both before and after treatment. A urine pregnancy test must be done before recruiting a female patient of childbearing age.

### Data management

In our study, all data are entered electronically using Oracle Clinical Remote Data Capture. Original study forms will be kept on file at the participating sites for a period of 3 years after completion of the study. At the end of the trial, the investigators, data managers and statisticians will perform a blind review and confirm the database before processing the data. Only the principal investigator will have access to the final trial data set. All patients’ personal information is classified and is used only in this study.

### Adverse event reporting

Adverse events will be recorded in detail using medical terminology throughout the study. Severe adverse events must be reported to both the institutional review board and the principal investigator within 24 hours. If there is a medical emergency, the unblinding procedures will be initiated by the principal investigator.

### Sample size calculation and statistical analysis

The sample size was determined using PEMS 3.1 with 90% power and alpha = 0.05 (two-sided). The mean duration of all symptoms for a common cold was estimated to be 7 days with a standard deviation of 4 days [15]. To demonstrate a reduction in the duration of all symptoms by 2 days, which is defined as clinically relevant, the sample size needs to be 85 cases for each group. Considering the potential loss of patients, the number of participants recruited must be 96 per group. Therefore, a total of 288 patients will be recruited in this trial.

The statistical analysis for this study will be performed by an independent professional statistician using PEMS 3.1 in a separate location. An intent-to-treat analysis for the patients, who have received treatment at least once, will be carried out. Missing data will be adjusted using the last observation carried forward method. The per-protocol analysis will be restricted to participants who strictly follow the protocol and complete the study. The safety analysis will be conducted only for randomized subjects who have completed at least one study visit. Pearson’s chi-square test or Fisher’s exact test will be performed on categorical variables, Student’s *t*-test on continuous normally distributed variables and the Wilcoxon rank sum test on non-normal variables. Due to the multicenter design, the Cochran–Mantel–Haenszel test stratified by clinical centers and analysis of covariance adjusted for clinical center and baseline will be used in this study. The statistical significance level will be set at *P* < 0.05 and 95% confidence interval will be calculated.

## Discussion

To date, there are still no effective therapeutic options available to treat the common cold. It seems that a vaccine may be a good solution. However, vaccines only work on certain viruses. Besides, since diverse viral serotypes cause the common cold, vaccine preparation is very difficult. Therefore, recent studies have mainly focused on symptomatic management. TCM is widely used in China to treat the common cold and many studies have shown that a TCM formulation might be better in reducing symptoms than a placebo [16-19]. However, the quality of many of these reported studies is of great concern [20]. A number of high-quality clinical trials are needed before any Chinese herbal preparation can be recommended for the common cold [21]. For this reason, the study protocol was designed according to the Consolidated Standards of Reporting Trials statement [22]. Quality controllers will pay regular visits to each center, recheck CRFs and monitor the research data throughout the trial. Moreover, investigators in different centers are all required to follow the standard operating procedures. To our knowledge, previous studies have only focused on wind-cold syndrome and wind-heat syndrome. Therefore, this will be the first multicenter, double-blind, double-dummy, randomized and placebo-controlled trial designed to treat CCSDS in an adult population from China. It will establish the basis for a scientific and objective assessment of the efficacy and safety of *Baoji Tablets* for treating CCSDS, and provide evidence for a phase III clinical trial.

## Trial status

This trial is currently recruiting participants.

## Abbreviations

CCSDS: common cold with summer-heat and dampness syndrome; CFDA: China food and drug administration; CRF: case report form; PEMS: Package for encyclopaedia medical statistics; PSSR: percentage of symptom score reduction; TCM: traditional Chinese medicine.

## Competing interests

The authors declare that they have no competing interests. Neither the funding agency nor any outside organization has a role in study design or manuscript preparation.

## Authors’ contributions

R-ZF, J-QL and BM contributed to the design and development of the study protocol. R-ZF prepared the initial draft of the manuscript. J-QL, AKJ and JDM contributed to and revised the manuscript. BM was the general supervisor for this research. All authors critically reviewed the content and approved the final version.
